# Effect of Botulinum Toxin Injection on EMG Activity and Bite Force in Masticatory Muscle Disorder: A Randomized Clinical Trial

**DOI:** 10.3390/toxins14080545

**Published:** 2022-08-10

**Authors:** Victoria Sitnikova, Antti Kämppi, Olli Teronen, Pentti Kemppainen

**Affiliations:** 1 Department of Oral and Maxillofacial Diseases, Faculty of Medicine, University of Helsinki, 00280 Helsinki, Finland; 2 Dental Clinic, Private Practice Mehiläinen, 00100 Helsinki, Finland; 3 Oral and Dental Centre, Helsinki University Central Hospital (HUCH), 00290 Helsinki, Finland

**Keywords:** temporomandibular disorders, masticatory muscle disorder, myofascial pain, chronic pain, botulinum toxin type A

## Abstract

Botulinum toxin type A (BoNT-A) is increasingly used in treating masticatory muscle pain disorder; however, safe doses and reinjection intervals still need to be established. The purpose of this randomized clinical trial was to evaluate the degree and duration of the impairment of masticatory muscle performance. Fifty-seven subjects were randomly divided into two groups: one of which received BoNT-A first (*n* = 28) while the other received saline first (*n* = 29), with the cross-over being in week 16, and a total follow-up period of 32 weeks. A total dose of 50 U of BoNT-A was injected in the masseter and temporal muscles bilaterally. Electromyographic (EMG) activity and bite forces were assessed. A significant reduction in EMG activity was observed up to week 18 (*p* ≤ 001), with total recovery at week 33. A significant reduction in maximum bite force was observed up to week 11 (*p* ≤ 005), with total recovery at week 25. In conclusion, when treating masticatory muscle pain disorder with 50 U of BoNT-A, a reinjection interval of 33 weeks can be considered safe since the recovery of muscle function occurs by that time.

## 1. Introduction

Masticatory muscle disorder is a common pain condition classified as temporomandibular disorder (TMD) [1]. It can be subclassified into myalgia, myofascial pain, and headache attributed to TMD. Noninvasive therapies, including behavior therapy, pharmacotherapy, physical therapy, and occlusal appliances are effective in most cases. Still, approximately 10% of TMD patients develop a disorder associated with chronic orofacial pain [2,3]. For these patients, injections of botulinum toxin A (BoNT-A) have been suggested as a treatment of choice given its relaxing and analgesic effect. The use of BoNT-A has significantly increased over recent years. However, its safety when injected systematically remains unclear.

Most of the studies concerning botulinum toxin for treating TMD concentrate on the drug’s effect on pain findings [4,5,6]. Some temporary and mostly insignificant adverse effects had been reported by the patients according to several studies, such as pain at the injection site, bruising, muscle weakness, and undesirable muscle paralysis [7,8]. In virtually all studies, they are reported as qualitative results, limited to self-reporting, concern the single injection of the current study, and are caused by the puncture itself. However, the objective evaluation of the drug’s effect on muscle and surrounding structures should also be taken into consideration since it is a toxin that causes muscle paralysis and the accumulation of physiological changes due to systematic injections might lead to unfavorable long-term side effects harming the masticatory system.

It is generally thought that muscle function is restored in 3 to 6 months after the injections [9,10]. However, in studies where muscle functional effect was evaluated using electromyography (EMG), the effect of the drug was present for up to 12 months [11]. The studies evaluating bony changes corroborate the drug’s long-lasting hidden effect [12,13]. Permanent damage to neuromuscular function has also been considered [14]. The effect is likely to be cumulative if the structures are not allowed to recover before the repeated injections [15]. Thus, understanding the muscle recovery rate is important to establish safe intervals for reinjections.

In this randomized controlled trial, the recovery of muscle function after a single injection of 50 U of BoNT-A was quantitively evaluated. The parameters used in this research are EMG and bite force.

## 2. Results

### 2.1. Patients

**Figure 1:**
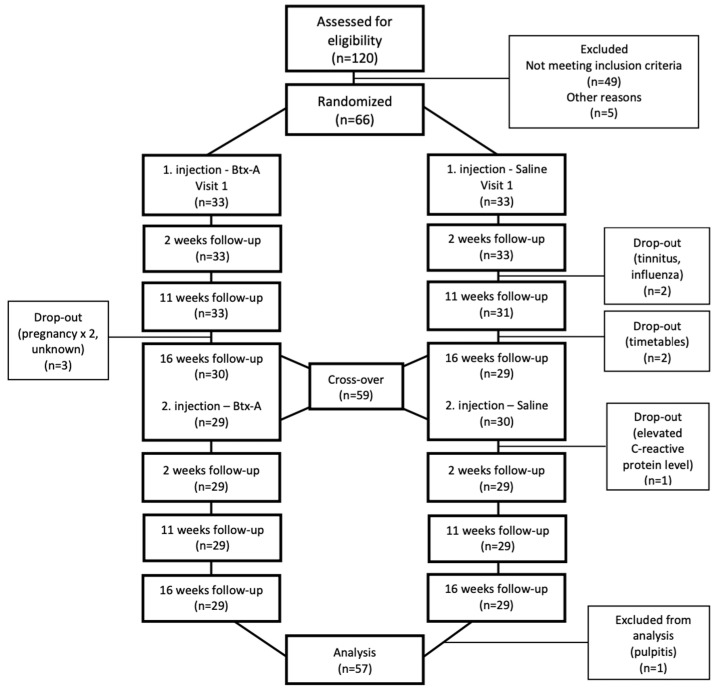
CONSORT diagram for participant enrollment.

The process from patient recruitment to analysis is illustrated as a flow diagram (Figure 1). Twenty-eight participants included in the analysis received BoNT-A first (the BS group) and twenty-nine received saline first (the SB group). The demographic and diagnostic characteristics of the subjects appear in Table 1 and Table 2. Most patients were female, (82.5%) with a mean age of 38.2 (range 22 to 64 years). Educational level was high, with 26.3% having graduated from the university of applied science and 38.6% from the university. The majority were employed.

Fifty-two patients (91.2%) were diagnosed with TMD-related myalgia, of which, twenty-one (38.2% of all patients) were diagnosed with myofascial pain with a referral. Twenty-four patients (42.1%) had headaches attributed to TMD. Three patients were diagnosed with right arthralgia and eight with left arthralgia. Almost half of the patients (47.4%) had suffered from pain symptoms for at least ten years. Twenty-three patients (40.4%) reported persistent pain symptoms and thirty-one (54.4%) reported recurrent pain symptoms.

### 2.2. Electromyography (EMG)

**Figure 2:**
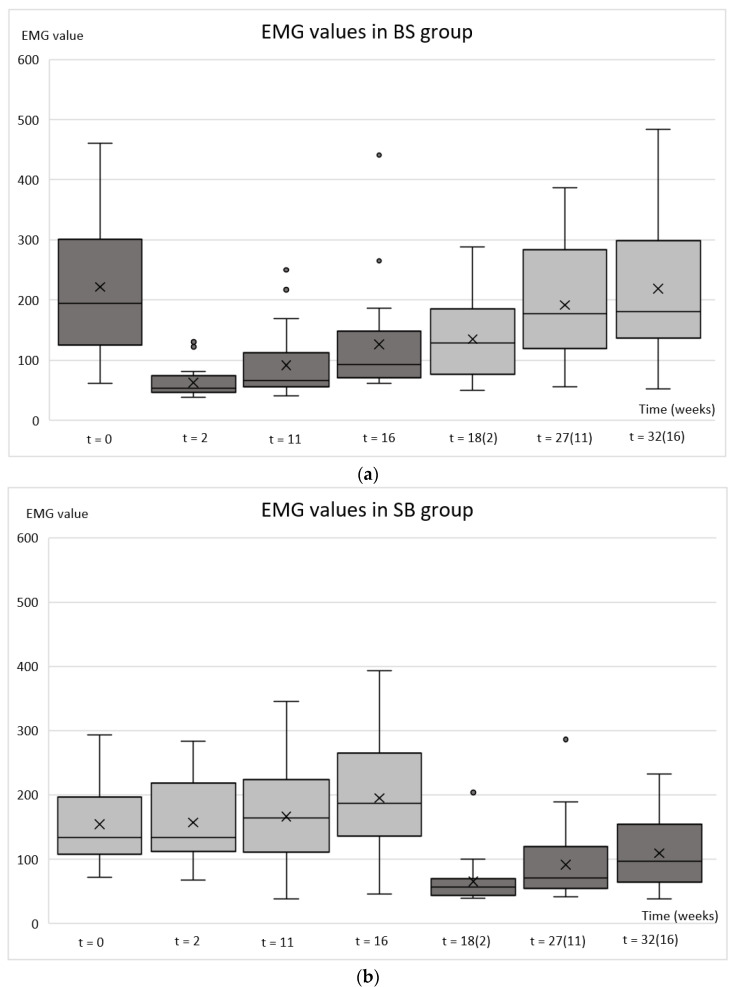
Box plots of the average electromyography (EMG) values of the masseter muscle after botulinum toxin and saline injections. The BS group (**a**) botulinum toxin injected at the baseline and saline at the 16-week follow-up; the SB group (**b**) saline injected at the baseline and botulinum toxin at the 16-week follow-up. The week calculated from the second injection is marked in brackets. For each box plot, maximum and minimum, 25th and 75th percentiles, median (solid line inside the box), mean (cross inside the box), and outliers (dots) are presented.

The greatest decrease in EMG activity was observed two weeks after the BoNT-A injections (Figure 2). The effect of the drug attenuated continuously up to week 32.

The mean EMGmax values over time for the BS group were 222 mV (day 0), 62 mV (week 2), 92 mV (week 11), 126 mV (week 16), 135 mV (week 18, i.e., 2 weeks after the second injection), 191 mV (week 27, i.e., 11 weeks after the second injection), and 219 mV (week 32, i.e., 16 weeks after the second injection) (Figure 2a). The mean EMGmax values over time for the SB group were 155 mV (day 0), 158 mV (week 2), 170 mV (week 11), 195 mV (week 16), 65 mV (week 18, i.e., 2 weeks after the second injection), 93 mV (week 27, i.e., 11 weeks after the second injection), and 112 mV (week 32, i.e., 16 weeks after the second injection) (Figure 2b).

**Figure 3:**
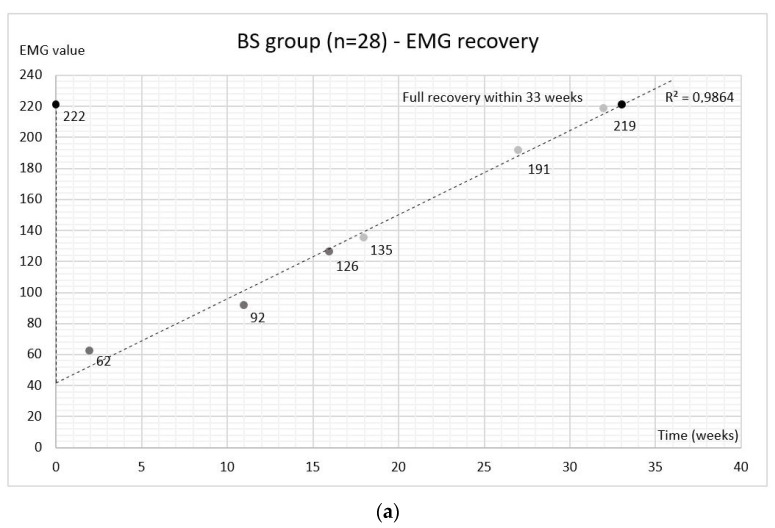

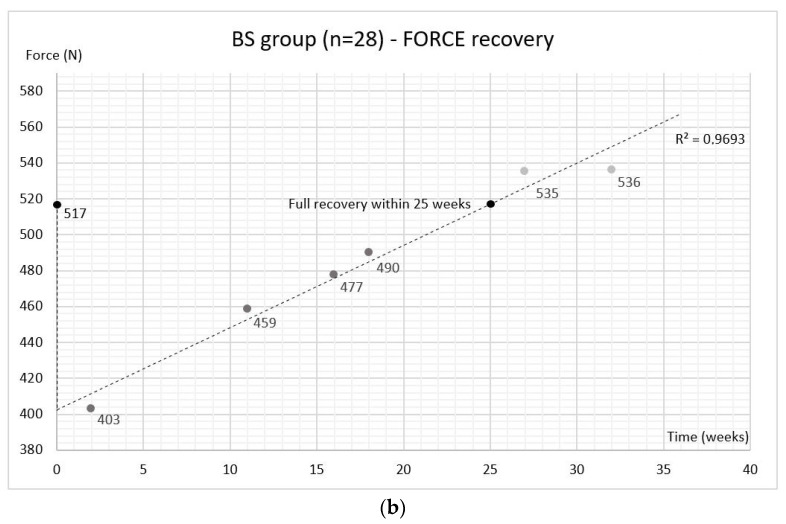
Regression lines for EMG (**a**) and bite force (**b**) mean values after botulinum toxin injection. BS group: botulinum toxin injected at the baseline and saline at the 16-week follow-up.

Comparing EMGmax values within the BS group between time-points showed a highly significant decrease (*p* ≤ 0.001) in EMGmax values on follow-up weeks 2, 11, 16, and 18 compared to the values before the BoNT-A injection (Table 3). EMGmax values were decreased by 72% at the 2-week follow-up, 59% at the 11-week follow-up, 43% at the 16-week follow-up, 39% at the 18-week follow-up, 14% at the 27-week follow-up, and 1% at the 32-week follow-up. The linearity was observed in the recovery of EMGmax values and full recovery was expected by week 33 based on the forecast function (Figure 3a).

Comparing the EMGmax values within the SB group between time-points showed an increase in EMGmax after saline injection (Figure 2b). The difference was highly significant at the 16-week follow-up compared to the baseline (*p*_0–16_ = 0.005) (Table 3). The increase in EMGmax values was also noticeable at the 11-week follow-up, even though statistically non-significant (*p*_0–11_ = 0.079). A highly significant decrease (*p* ≤ 0.001) in EMGmax values was observed after the second injection in follow-up weeks 18, 27, and 32 (i.e., 2, 11, and 16 after the BoNT-A injection). This is consistent with the findings of the BS group.

There were no great differences in EMGrest values over time either in the BS or SB groups; the values ranged from 38 mV to 41 mV.

### 2.3. Bite Force

**Figure 4:**
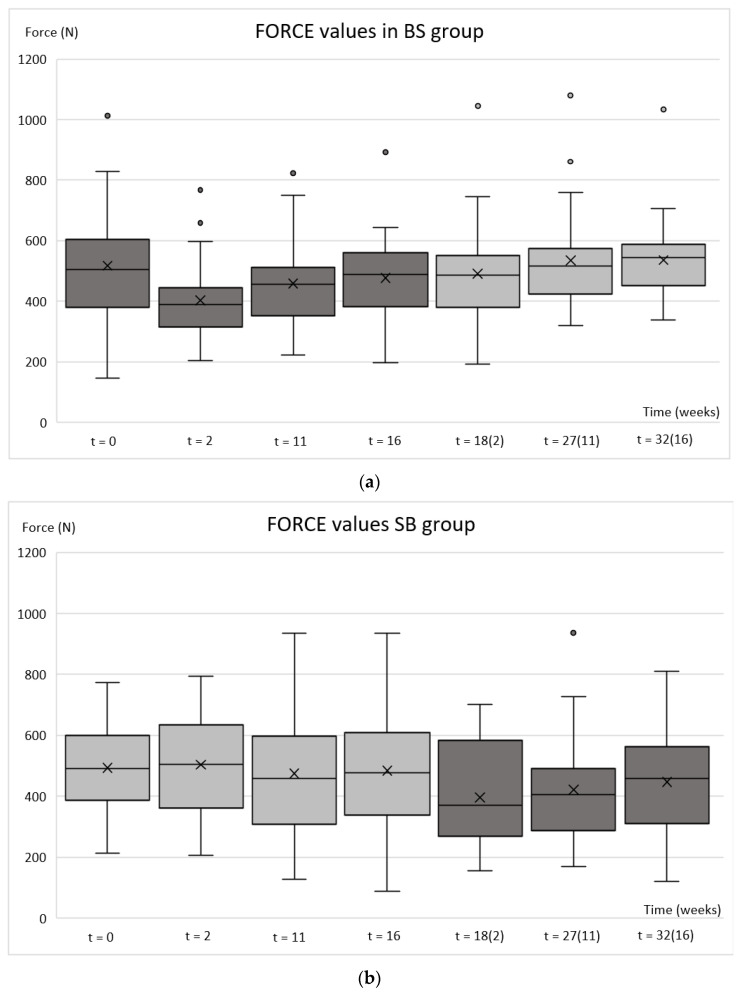
Box plots of bite force values after botulinum toxin and saline injections. BS group (**a**) botulinum toxin injected at the baseline and saline at the 16-week follow-up; SB group (**b**) saline injected at the baseline and botulinum toxin at the 16-week follow-up. The week calculated from the second injection is marked in brackets. For each box plot, maximum and minimum, 25th and 75th percentiles, median (solid line inside the box), mean (cross inside the box), and outliers (dots) are presented.

Regarding bite force, the average decrease of 114 N was achieved with 50 U of BoNT-A. As with the EMG, the lowest value was obtained two weeks after the drug injection. The effect of the drug attenuates continuously up to week 27 (Figure 4).

The mean maximum bite force values over time for the BS group were 517 N (day 0), 403 N (week 2), 459 N (week 11), 477 N (week 16), 490 N (week 18, i.e., 2 weeks after the second injection), 535 N (27 weeks, i.e., 11 weeks after the second injection), and 536 N (32 weeks, i.e., 16 weeks after the second injection) (Figure 4a). The mean maximum bite force values over time for the SB group were 497 N (day 0), 508 N (week 2), 478 N (week 11), 486 N (week 16), 399 N (week 18, i.e., 2 weeks after the second injection), 427 N (27 weeks, i.e., 11 weeks after the second injection), and 453 N (32 weeks, i.e., 16 weeks after the second injection) (Figure 4b).

Comparing the maximum bite force values within the BS group between time-points showed a significant decrease at the 2 (*p*_0–2_ ≤ 0.001) and 11 week (*p*_0–11_ = 0.002) follow-up compared to baseline (Table 3). Bite force values were decreased by 22% at the 2-week follow-up, 11% at the 11-week follow-up, 8% at the 16-week follow-up, and 5% at the 18-week follow-up. Compared to the baseline, 3% higher values at the 27-week follow-up and 4% at the 32-week follow-up were observed (Figure 4a). As with EMGmax values, the linearity was observed in the recovery of bite force and full recovery occurred at 25 weeks after BoNT-A injections based on the forecast function (Figure 3b).

When maximum bite force values were compared within the SB group, no significant changes in values were observed after the saline injection. However, a significant decrease in bite force was noticed 2 weeks and 11 weeks after the BoNT-A injections (*p*_0–18_ ≤ 0.001 and *p*_0–27_ = 0.025) (Table 3).

### 2.4. Harm

A total of 3 patients out of 59 reported harm after the BoNT-A injections. One patient reported smile asymmetry ten days after the injections, indicating that mimic muscles might have been involved. The recovery occurred within eight weeks. One patient reported the onset of migraine a few days after the BoNT-A injections and another reported tinnitus and pain symptoms in both ears, with the symptoms being gone at two weeks’ follow-up.

## 3. Discussion

BoNT-A has gained a lot of interest in treating masticatory muscle pain conditions. Along with its analgesic effect, BoNT-A also causes structural and functional changes in muscle tissue, which are considered adverse reactions [7]. There are only a few studies evaluating the recovery of muscle along with pain symptoms [16,17,18]. EMG and bite force are both important parameters in evaluating masticatory muscle performance and the measurement methods are reliable, convenient, and safe. In addition, decreased EMG implies that neuromuscular junctions have not been restored, which directly reflects BoNT-A’s denervation effect [14]. Our study showed that the recovery of EMG occurs by 33 weeks and bite-force by 25 weeks after a moderate dose of BoNT-A.

The present study supports previous findings concerning the significant decrease in EMG values after BoNT-A injections. However, the recovery course and time differ slightly. In our study, the maximum decrease of 72% of EMG of the masseter muscle was observed two weeks after the injection of 16.7 U into the masseter muscle and a significant reduction was still present 18 weeks later. The effect of BoNT-A was also noticeable 27 weeks after the injections with a total recovery taking 33 weeks. De la Torre Canales et al. reported about an 80% decrease in EMG values 28 days after the injections of 30 U of BoNT-A, with a total recovery after 180 days [16]. Compared to our study, the recovery of EMG was achieved earlier with a bigger dose. Kurtoglu et al. reported significantly lower EMG activity on day 14, but not on day 28 after the injection of 30 U of BoNT-A [18]. Such a rapid recovery is somewhat at odds with our findings.

Interestingly, EMGmax values increased slightly after saline injections in the SB group. In this blinded research setting, the subject might test the effect of the treatment by testing their muscle function, which might further increase the tonus of the jaw-closing muscles. This effect of the intervention itself is clinically not significant. However, this finding might indicate that EMG values would still be decreased 33 weeks after the drug injection when the effect of the intervention itself is eliminated.

Regarding maximum bite force, a 22% reduction was observed in our study two weeks after the total dose of 50 U was injected into the masseter and temporal muscles bilaterally. The values were still lower 18 weeks after the drug injections, but the difference was not significant, and total recovery was achieved by 25 weeks. No significant changes in bite force were noticed after saline injections. This means that the finding of this study is due to the drug itself and not to the placebo or intervention. In a previous study using 100 U for the masseter muscles (50 U each), bite force decreased approximately 40% three months after the injections, and the values were still decreased by approximately 35% six months after the treatment [19]. Jadhau et al. also reported the lowest decrease three months after injections with 100 U of BoNT-A, the values being still lower six months later [20]. Combining these results with ours might suggest that the decreased bite force, as well as recovery rate, are dose-dependent. In a study using 42 U for the masseter muscles (21 U each) a decrease of approximately 50% was observed 10 days after the injections, with a significant reduction still present 20 weeks after the treatment [21]. Obviously, as with EMG activity, BoNT-A injection decreases bite force according to any research. However, the differences in the injection areas, doses, and timing of follow-up visits mean that the results are not directly comparable.

It is known that decreased muscle-derived force and muscle activity cause bone loss [22]. Changes in bone volume and density are also reported in studies that evaluate the effect of BoNT-A injections [12,23]. The decreased values of EMG and bite force reported in this study may predict the presence of latent adverse effects beyond the injected muscle. Alongside the mechanical theory, biochemical interactions between muscle and bone tissue have recently been the subject of interest [24]. The masticatory system, which is made up of bones, joints, ligaments, teeth, and muscles, is a highly refined unit. There is complex molecular crosstalk between components and altering the function of one component may lead to the malfunction of another component. The biomechanical and biochemical interactions are particularly important in the development phase of masticatory structures, meaning that the injections are contraindicated in growing individuals.

In our study, most patients were female (82.5%), which is partly explained by the fact that TMD is more prevalent in women than in men [25,26]. Of the sixty-six randomized patients, nine dropped out during the study. An interesting fact is that all those who left the study voluntarily received saline first and did not wait for the BoNT-A injection. This might be explained by patients’ expectations of a quick fix when seeking medical help and not deriving benefit from the placebo. The high education level of the subjects of our research might reflect the patient’s financial situation and thus the possibility of applying for private dental care. However, the results of the previous studies regarding associations between TMD and education level are not conclusive [27,28].

The randomization, blinding, and placebo control used in our study are gold standards to evaluate the effects of medical intervention. To reduce the heterogeneity of the subjects included in the study, a standardized examination protocol of TMD-patients (DC/TMD), as well as history and anamnesis questionnaires, were used. However, because of the randomization, the differences in the initial scores between groups could not be avoided. In this research, the initial EMGmax values were higher in the BS group than in the SB group, which, however, has no influence when the values are compared within one group.

The results reported herein should be considered in light of some limitations. First, the recovery rate reported based on the BS group findings only might be influenced by the saline injections that were performed at the 16-week follow-up. However, changes caused by saline compared to changes caused by BoNT-A, were rather insignificant, as was seen in the SB group. Second, the decreased muscle activity and bite force hardly reflect the beneficial gain of the treatment. The analgesic effect of BoNT-A is thought to be the main mechanism of action when treating pain conditions and is independent of its neuromuscular effect [17]. Masticatory muscle pain is related more to patient psychological status than muscle activity [29]. There is also evidence that occlusal forces do not correlate with TMD symptoms [30]. The findings of this research do not give information on BoNT-A indications when treating pain symptoms. While the decision to inject is always made according to the symptoms and diagnosis, there is still no consensus on the indications and dose appropriateness. Earlier studies, with doses ranging from 80 to 200 U, proved that BoNT-A’s pain-relieving effect can last up to 4–6 months [5,6,16]. Up to six months of analgesic effect from BoNT-A had also been observed in the treatment of trigeminal neuralgia [31]. De Lima et al. studied the effect of 20 U on TMD pain findings, concluding that a low dose can be effective for controlling chronic pain, but this outcome was short-lived, with pain scores reaching the baseline within 30 days [32]. Whether a total dose of 50 U can be an optimal one with acceptable adverse effects in relation to therapeutic benefit remains a subject of interest. Third, even though BoNT-A injections impair skeletal muscle function, the clinical importance of this is still unclear. Side effects should be evaluated after multiple treatments with longer follow-up periods. So far, low doses of BoNT-A are suggested to be safer because the degree and duration of the adverse effects are dose-dependent [16]. In addition, according to some studies, bigger doses are not likely to bring more therapeutic benefits when treating pain conditions [16,33]. In further clinical trials, the beneficial effect of a moderate dose of BoNT-A, such as 50 U, should be evaluated.

## 4. Conclusions

We conclude that the recovery of muscle function occurs 33 weeks after the injection of 50 U of BoNT-A when treating masticatory muscle pain disorder. Thus, that period can be considered safe for reinjections. Further studies should evaluate the effect of this dose of the drug on pain findings and its ratio to adverse effects.

## 5. Materials and Methods

### 5.1. Patients

Voluntary subjects from several mainly private dental practices were recruited between May 2019 and April 2021. Patients could sign up for the study based on their own assessment of the need for treatment of orofacial pain, regardless of whether the condition had previously been treated conservatively or not. They were subsequently screened for specific inclusion and exclusion criteria. The total number of participants included in the study was 57, predominantly female. The inclusion criteria were age > 18, diagnosis of myalgia, myofascial pain, or headache attributable to TMD according to Diagnostic Criteria for Temporomandibular Disorders (DC/TMD) with persistent or bothersome recurrent pain for at least a year and present during the past month. The history questionnaire of the Research Diagnostic Criteria for Temporomandibular Disorders (RDC/TMD) was used to evaluate pain chronicity and frequency. Most of the patients had undergone conservative TMD treatment such as an occlusal splint or physiotherapy but without complete relief of symptoms. Exclusion criteria for the study were the following: previous injections of BoNT-A for the treatment of TMD, arthrosis, arthritis, neuromuscular pathologies, major psychiatric disorders, pregnancy, and lactation. After the end of the study, the patient’s treatment was continued in accordance with Finnish Current Care Guidelines for TMD [34].

### 5.2. Experimental Protocol

This study was conducted as a crossover trial. Participants were randomly divided into two groups following simple randomization procedures: one which received BoNT-A first (the BS group) and the other which received saline first (the SB group). The second injection was performed after the washout period of 4 months (Figure 1). The patients were told they would receive both saline and BoNT-A once, but the order of the injections was unknown to them and the examiners. A random number table was used to allocate the participants. Randomization was performed by an investigator with no clinical involvement in the trial. Insulin syringes were filled either with BoNT-A dissolved in saline or saline only. The examiners received filled syringes marked with the code only. All syringes were similar in appearance. The codes of the substance injected were decoded and the data collected was analyzed using the patient’s ID once the clinical part of the research was over.

Three examiners enrolled the participants and assigned them to interventions. The same examiners conducted the clinical part of the study including injections, patient examination, and measurements. The examiners were taught the injection techniques by a maxillofacial surgeon experienced in injections. In accordance with the DC/TMD guidelines, the competence of the examiners is level two, which includes a two-day course on the use of the DC/TMD examination protocol. They were also instructed in using the EMG and bite force measurement devices by a person who used them in previous research [35,36].

The study lasted for 8 months for each subject and included seven follow-up visits. There were 28 patients in the BS group and 29 in the SB group (Figure 1). Follow-ups were performed at 2, 11, and 16 weeks after the first injection and the second injection, respectively (i.e., 18, 27, and 32 weeks after the first injection). EMG activity and bite force measurements were evaluated at baseline and each follow-up.

### 5.3. Injections

The injections were performed according to the techniques reported in previous research [13,37]. Each patient received a once-off treatment of 50 units of BoNT-A (Xeomin^®^, supplied by Merz Pharmaceuticals GmbH, Frankfurt, Germany). The powder was dissolved in 1.5 mL of sterile saline water. In total, 2/3 of the dose was injected into the bilateral masseter muscle (16.7 units each) and 1/3 into the bilateral temporalis muscle (8.3 units each). Three injections located 1–1.5 cm apart were applied along the inferior border of the masseter, and two injections located 2 cm apart were applied along the anterior part of the temporalis. In the placebo group, 1.5 mL of saline was injected. 

### 5.4. Treatment Outcomes

EMG and bite force measurements were performed with equipment that demonstrated reliability and repeatability in previous studies [35,36,37]. The examination was conducted in the same space and under the same conditions. Patients were in a sitting position, with the head supported and the Frankfort level parallel to the floor. On the intervention days, the measurements had been performed before the injections.

EMG parameters were assessed using a portable EMG device (ME 6000 recorder, Mega Electronics, Kuopio, Finland). The surface electrodes were positioned according to the anatomical landmarks on the masseter muscles. Inter-electrode distance recommendations of 30 mm were observed. The ground electrodes were placed on the chin. All electrode sites were cleaned by swabbing with 96% alcohol. For the analysis, the averaged EMG spectrum area data values calculated from a 2 s period were used.

Bite force was recorded with a device based on a quartz force transducer as a sensor. The sensor was built inside the 10 mm thick steel housing which was inserted along the dental arch so that the sensor was in the first molar region on the right site. To protect the teeth, the housing was covered with 2-mm-thick rubber plates on both sites. The maximal bite force value (N) could be seen on the screen of the bite force recorder.

Bilateral masseter muscle EMG activities and bite force were assessed in two serial recordings. A series of recordings consisted of a 30 s long exercise so that once every 10 s the patient should bite hard for 5 s following a 5 s relaxation. The total time of the recordings was 60 s with a few minutes break between the series. The patient was able to view the timing of the task on a computer screen. The EMGrest value used in the analysis was picked from the beginning of the recording. The EMGmax value used in the analysis was picked from the middle of the first series. The average of three maximal bite force measurements during a 60 s recording was calculated and used in the analysis.

### 5.5. Statistical Analysis

Parameters for power sample size estimation were determined by the authors as follows: test power 0.90 with a 0.05 significance level and effect size of 0.4. Considering these parameters, the appropriate sample size would have been 15 subjects. Other analyses of the research material and 50% dropout risk were also taken into account. Under these circumstances, the authors decided to recruit 120 subjects for the study in the first place. In the data used in this study, the observed test power was 1.0 with a significance level of <0.001 and an effect size of 0.51.

Mean values of EMG and bite force were calculated for each follow-up time. *p*-values were calculated for dependent mean variables using the Wilcoxon signed-rank test. Analyses were performed using SPSS version 28.0 (SPSS Inc., Chicago, IL, USA) and Office 365 Excel software 2108 (Microsoft Inc., Redmond, WA, USA).

## Figures and Tables

**Table 1:** Patient sociodemographic characteristics.

Sociodemographic Characteristics	% (*n*)
**Gender**	
Male (age 33.8 ± 8.5 [22–51])	17.5 (10)
Female (age 39.2 ± 10.5 [24–64])	82.5 (47)
All (age 38.2 ± 10.4 [22–64])	100.0 (57)
**Education**	
High school	5.3 (3)
Vocational school	24.6 (14)
Polytechnic	26.3 (15)
University	38.6 (22)
Not reported	5.3 (3)
**Occupation**	
Student	15.8 (9)
Employed (Entrepreneur)	75.4 (43)
Working at home	3.5 (2)
Not reported	5.3 (3)
**Marital status**	
Married	35.1 (20)
Cohabiting	24.6 (14)
Divorced	15.8 (9)
Never married	19.3 (11)
Not reported	5.3 (3)

**Table 2:** Patient diagnostic characteristics.

Diagnostic Characteristics	% (*n*)
**DC/TMD diagnosis**	
Myalgia	46.4(52)
Myofascial pain with referral	18.8 (21)
Headache attributed to TMD	21.4 (24)
Arthralgia right	2.7 (3)
Arthralgia left	7.1 (8)
Other *	3.6 (4)
**Pain chronicity**	
1 to <5 years	24.6 (14)
5 to <10 years	24.6 (14)
≥10 years	47.4 (27)
Not reported	3.5 (2)
**Pain frequency**	
Persistent	40.4 (23)
Recurrent	54.4 (31)
One-time	1.8 (1)
Not reported	3.5 (2)

* According to the History questionnaire of the Research Diagnostic Criteria for Temporomandibular Disorders patients have had pain in the face, jaw, temple, in front of the ear, or the ear in the past month. No TMD diagnosis was obtained based on clinical examination.

**Table 3:** Comparison of EMGmax and maximum bite force values within one group between time-points. BS group: botulinum toxin injected at the baseline and saline at the 16-week follow-up; SB group: saline injected at the baseline and botulinum toxin at the 16-week follow-up. The week calculated from the second injection is marked in brackets.

	*p* Values *					
	Time Range (Weeks)					
	0–2	0–11	0–16	0–18 (2)	0–27 (11)	0–32 (16)
EMG BS group	0.000	0.000	0.000	0.001	0.122	0.994
EMG SB group	0.582	0.079	0.005	<0.001	<0.001	<0.001
FORCE BS group	<0.001	0.002	0.167	0.109	0.838	0.885
FORCE SB group	0.084	0.922	0.945	<0.001	0.025	0.520

* Wilcoxon Sign Rank exact test.

## Data Availability

Data are available upon reasonable request.
